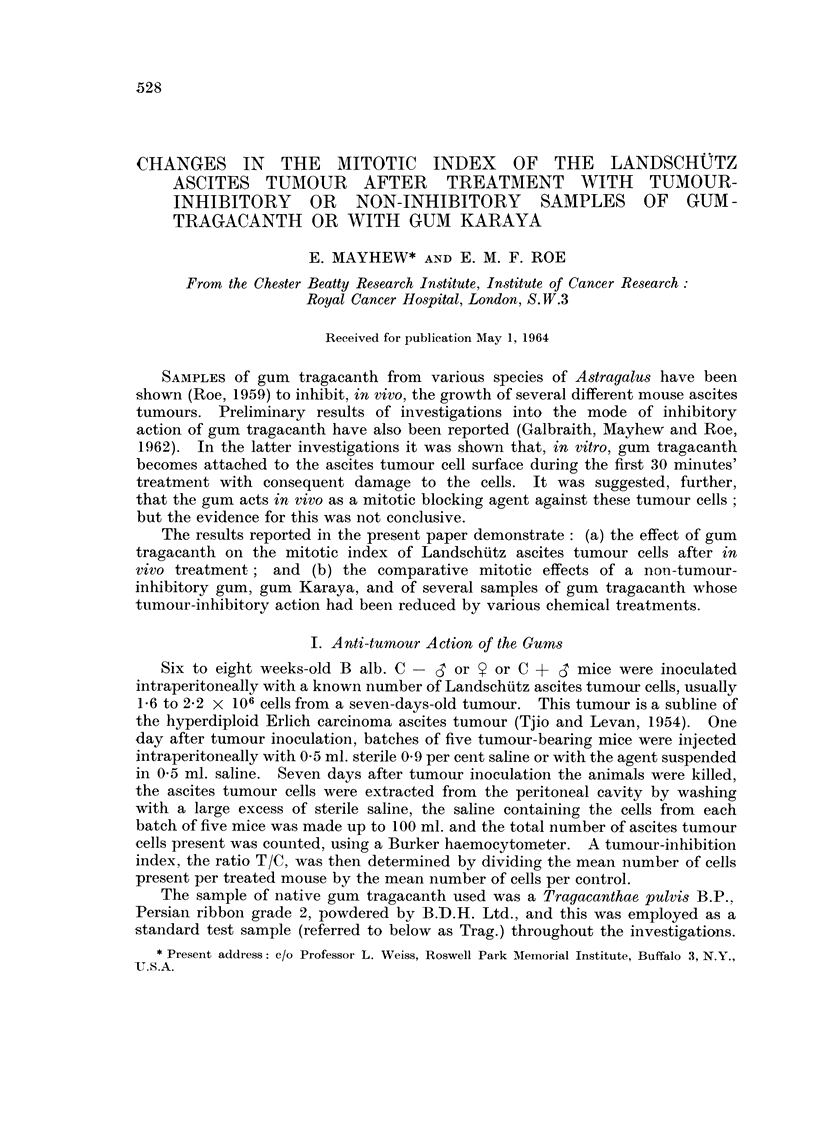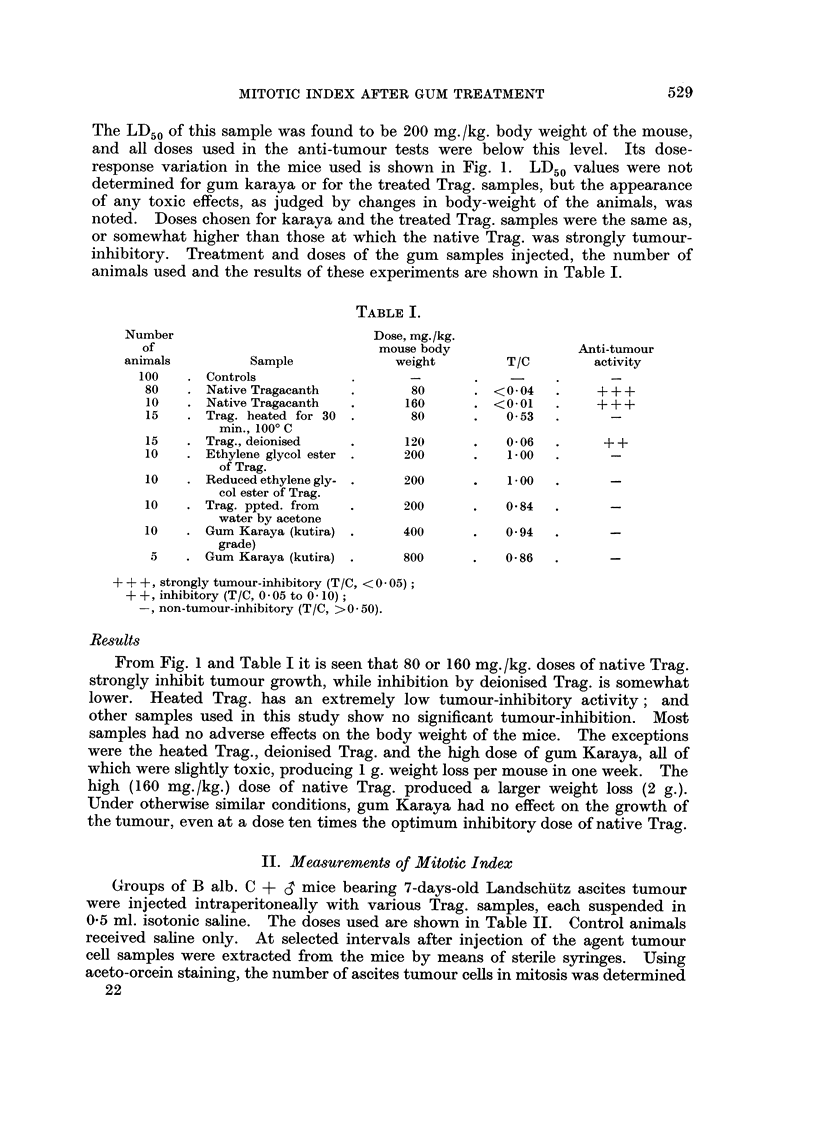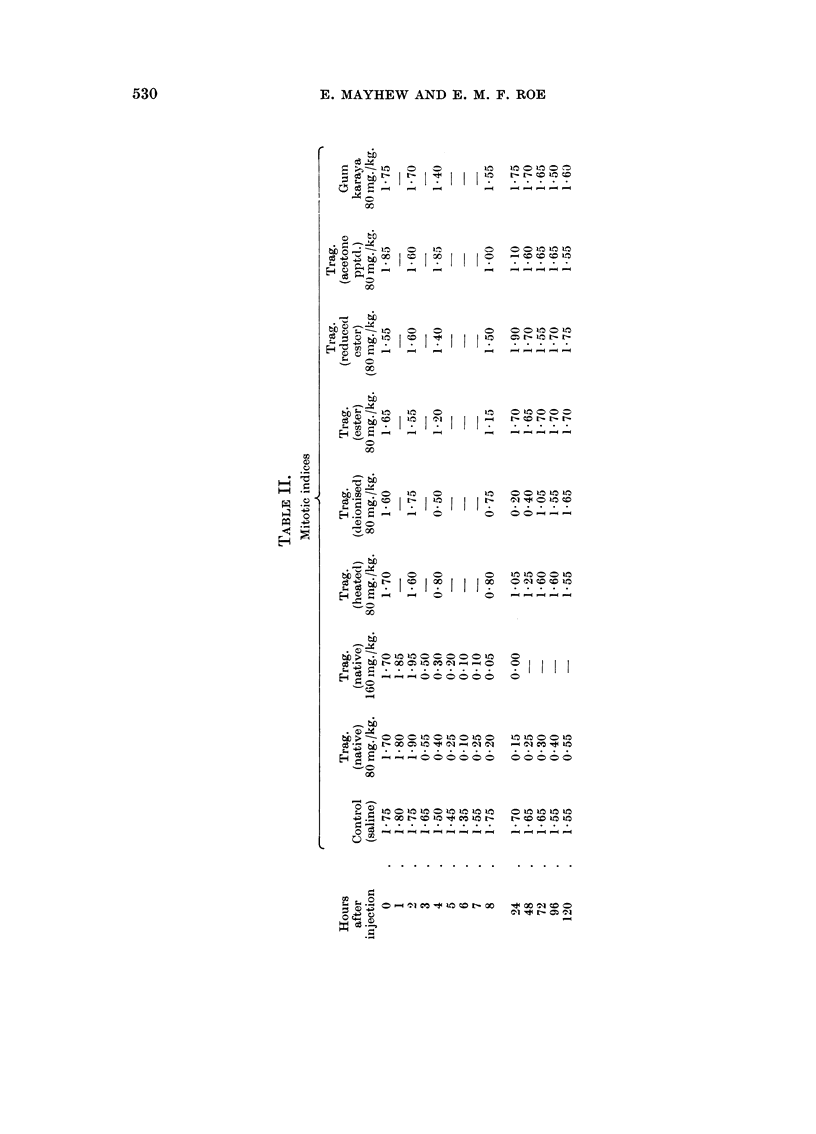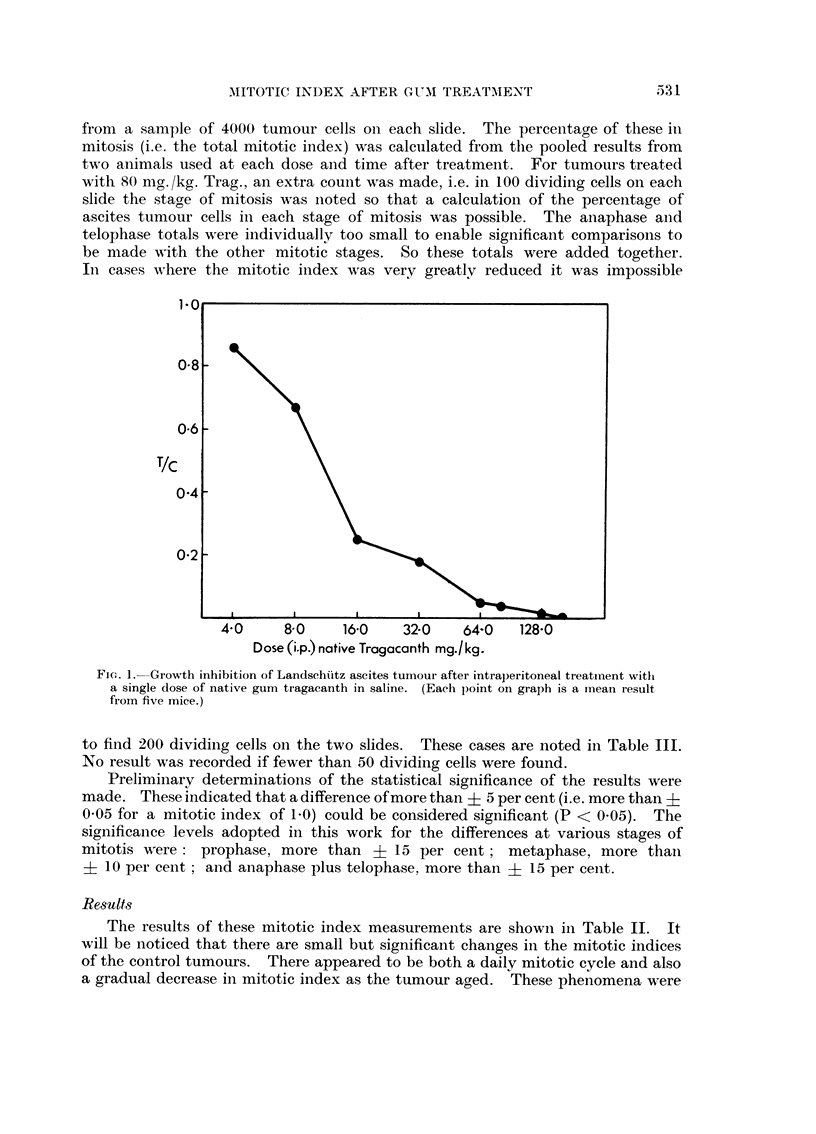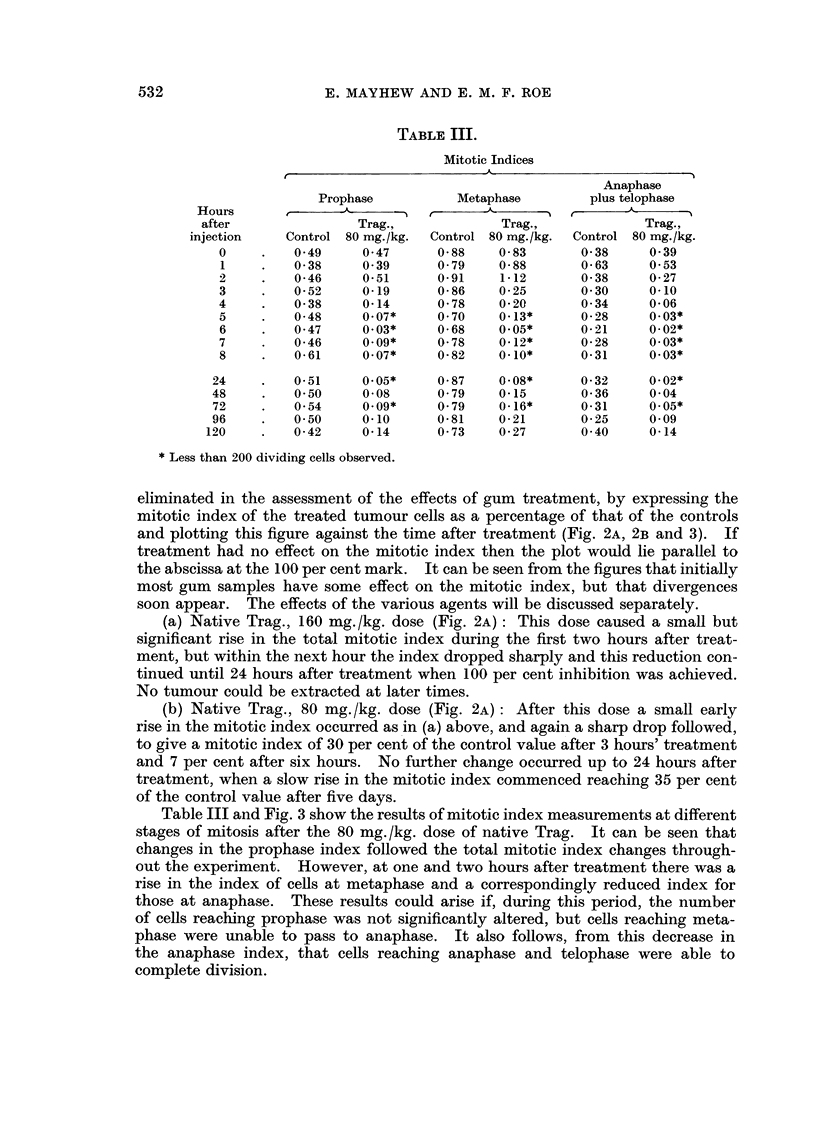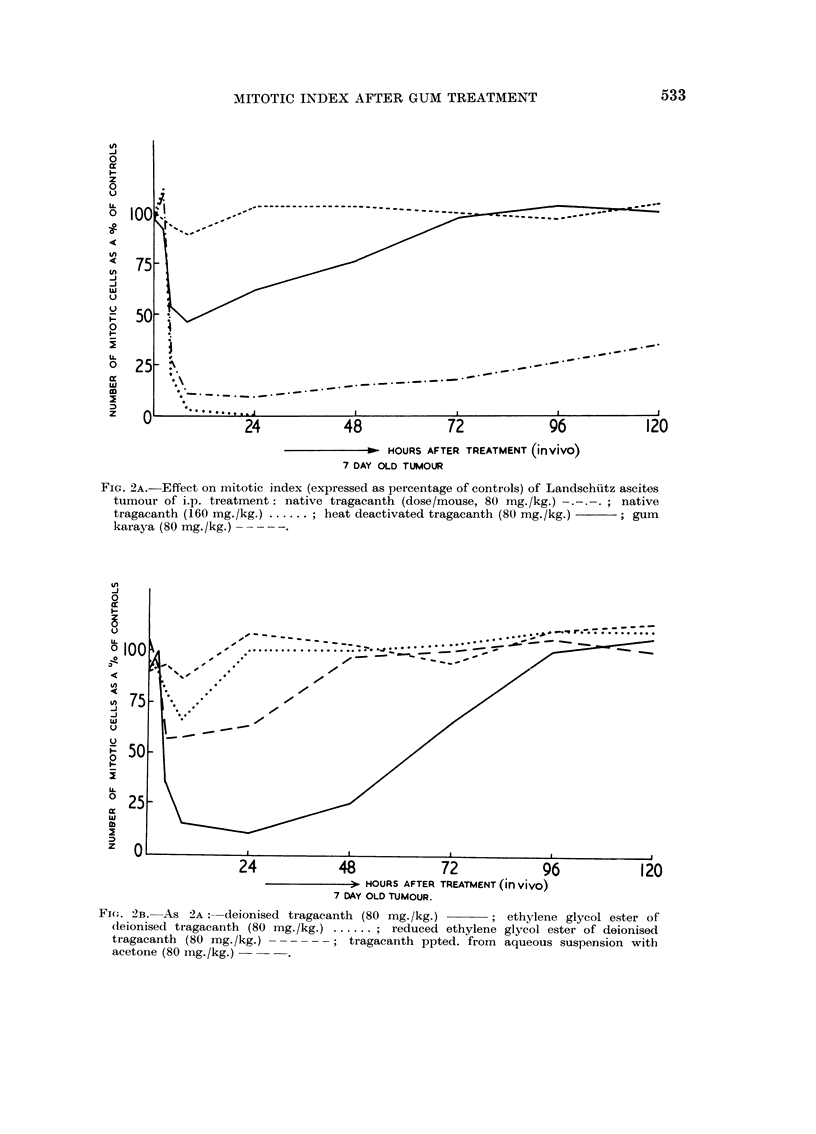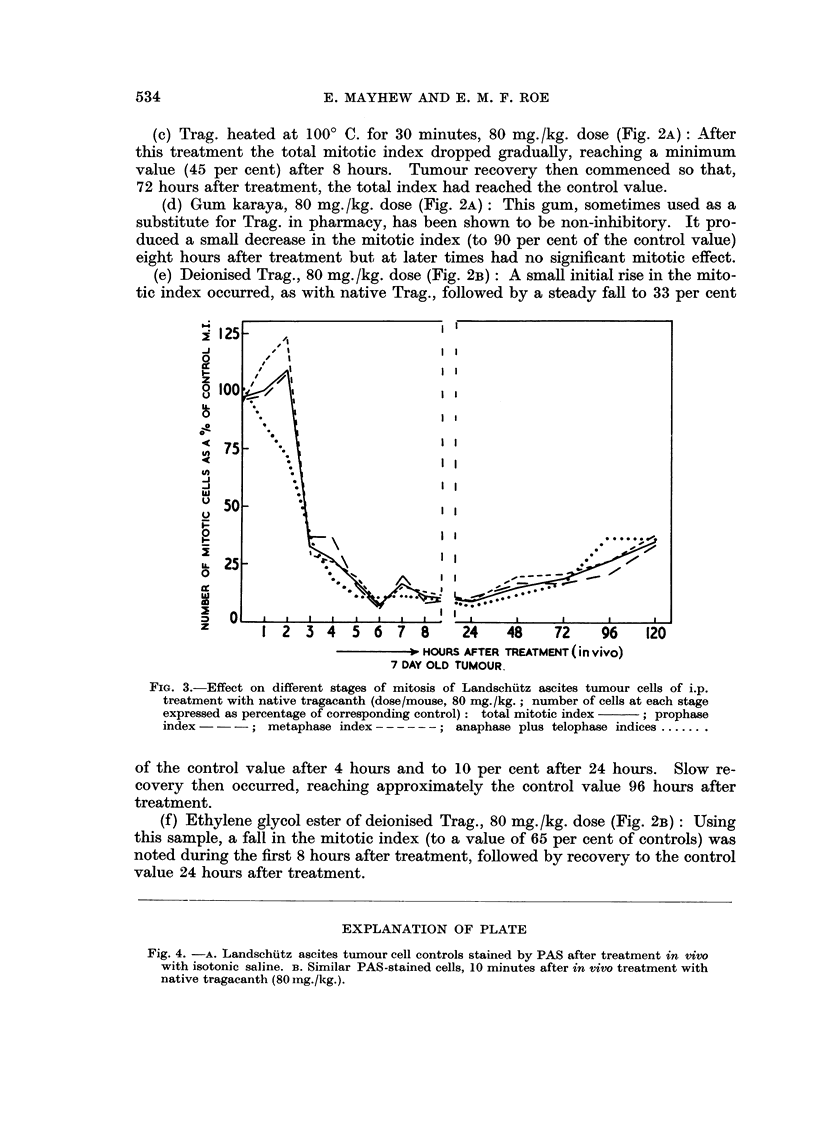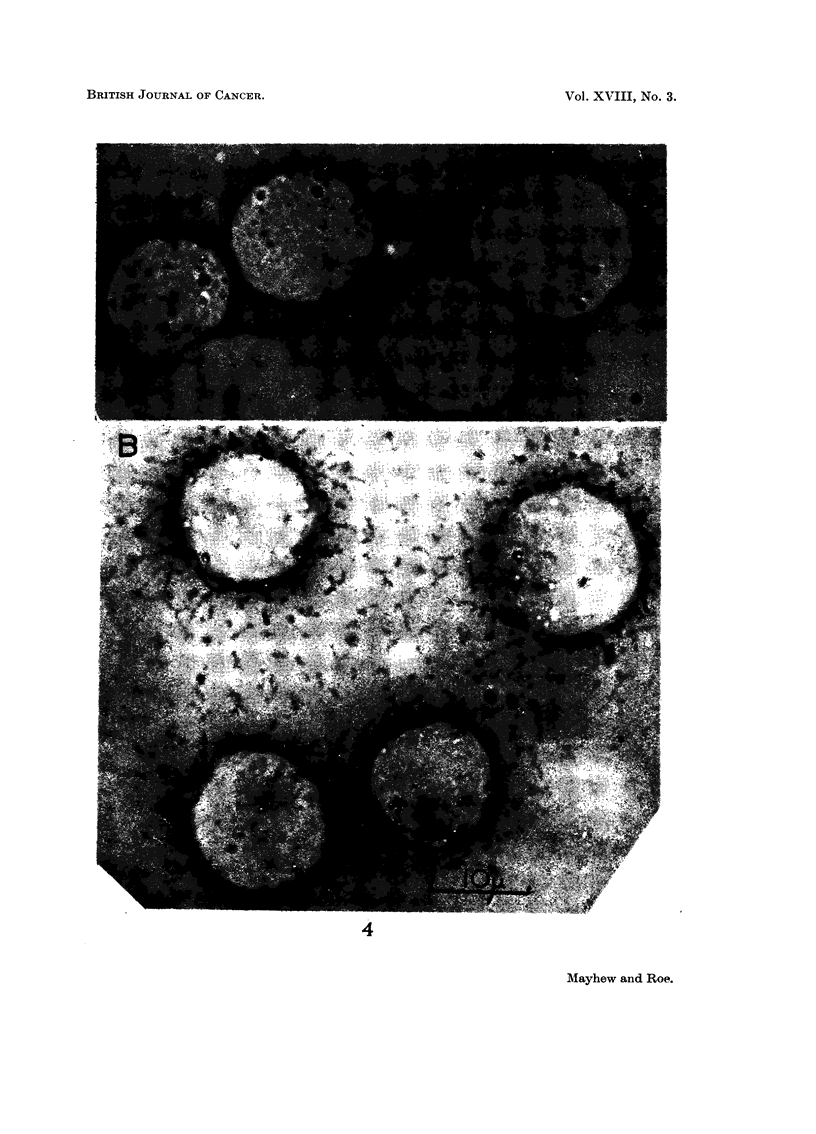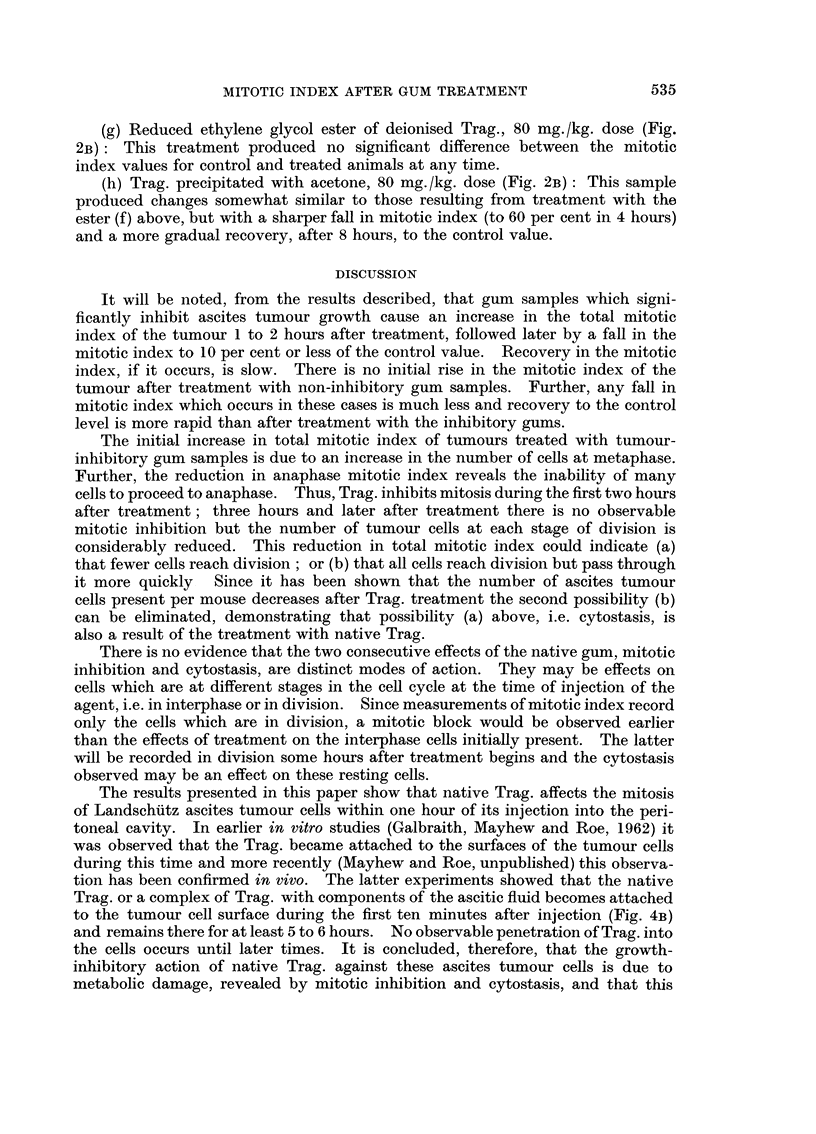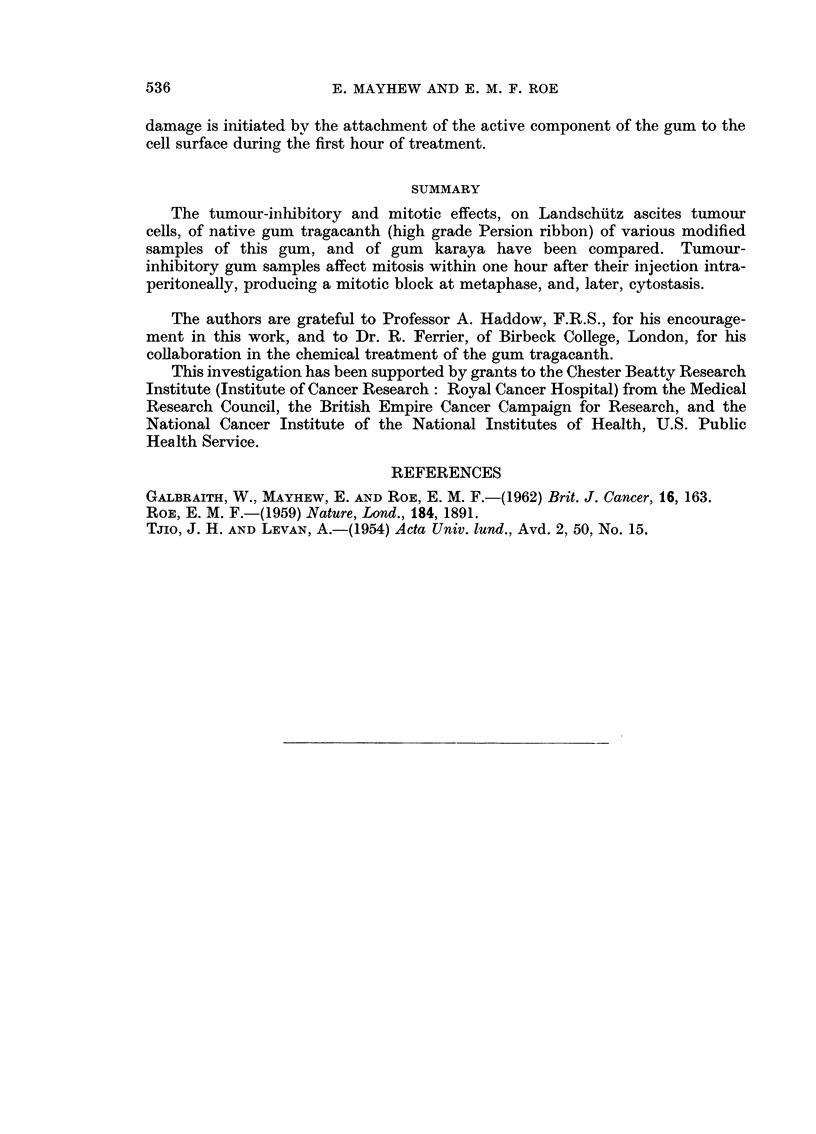# Changes in the Mitotic Index of the Landschütz Ascites Tumour after Treatment with Tumour-Inhibitory or Non-Inhibitory Samples of Gum Tragacanth or with Gum Karaya

**DOI:** 10.1038/bjc.1964.60

**Published:** 1964-09

**Authors:** E. Mayhew, E. M. F. Roe

## Abstract

**Images:**


### CHANGES IN THE MITOTIC INDEX OF THE LANDSCHÜTZ ASCITES TUMOUR AFTER TREATMENT WITH TUMOUR-INHIBITORY OR NON-INHIBITORY SAMPLES OF GUM-TRAGACANTH OR WITH GUM KARAYA

E. MAYHEW* AND E. M. F. ROE

*From the Chester Beatty Research Institute, Institute of Cancer Research : Royal Cancer Hospital, London, S.W.3*

Received for publication May 1, 1964

SAMPLES of gum tragacanth from various species of *Astragalus* have been shown (Roe, 1959) to inhibit, *in vivo*, the growth of several different mouse ascites tumours. Preliminary results of investigations into the mode of inhibitory action of gum tragacanth have also been reported (Galbraith, Mayhew and Roe, 1962). In the latter investigations it was shown that, *in vitro*, gum tragacanth becomes attached to the ascites tumour cell surface during the first 30 minutes' treatment with consequent damage to the cells. It was suggested, further, that the gum acts *in vivo* as a mitotic blocking agent against these tumour cells ; but the evidence for this was not conclusive.

The results reported in the present paper demonstrate : (a) the effect of gum tragacanth on the mitotic index of Landschütz ascites tumour cells after *in vivo* treatment ; and (b) the comparative mitotic effects of a non-tumour-inhibitory gum, gum Karaya, and of several samples of gum tragacanth whose tumour-inhibitory action had been reduced by various chemical treatments.

### I. *Anti-tumour Action of the Gums*

Six to eight weeks-old B alb. C − ♂ or ♀ or C + ♂ mice were inoculated intraperitoneally with a known number of Landschütz ascites tumour cells, usually 1·6 to 2·2 × $10^6$ cells from a seven-days-old tumour. This tumour is a subline of the hyperdiploid Erlich carcinoma ascites tumour (Tjio and Levan, 1954). One day after tumour inoculation, batches of five tumour-bearing mice were injected intraperitoneally with 0·5 ml. sterile 0·9 per cent saline or with the agent suspended in 0·5 ml. saline. Seven days after tumour inoculation the animals were killed, the ascites tumour cells were extracted from the peritoneal cavity by washing with a large excess of sterile saline, the saline containing the cells from each batch of five mice was made up to 100 ml. and the total number of ascites tumour cells present was counted, using a Burker haemocytometer. A tumour-inhibition index, the ratio T/C, was then determined by dividing the mean number of cells present per treated mouse by the mean number of cells per control.

The sample of native gum tragacanth used was a *Tragacanthae pulvis* B.P., Persian ribbon grade 2, powdered by B.D.H. Ltd., and this was employed as a standard test sample (referred to below as Trag.) throughout the investigations.

* Present address : c/o Professor L. Weiss, Roswell Park Memorial Institute, Buffalo 3, N.Y., U.S.A.

The $LD_{50}$ of this sample was found to be 200 mg./kg. body weight of the mouse, and all doses used in the anti-tumour tests were below this level. Its dose-response variation in the mice used is shown in Fig. 1. $LD_{50}$ values were not determined for gum karaya or for the treated Trag. samples, but the appearance of any toxic effects, as judged by changes in body-weight of the animals, was noted. Doses chosen for karaya and the treated Trag. samples were the same as, or somewhat higher than those at which the native Trag. was strongly tumour-inhibitory. Treatment and doses of the gum samples injected, the number of animals used and the results of these experiments are shown in Table I.

TABLE I.

| Number of animals | Sample | Dose, mg./kg. mouse body weight | T/C | Anti-tumour activity |
|---|---|---|---|---|
| 100 | Controls | — | — | — |
| 80 | Native Tragacanth | 80 | $<0 \cdot 04$ | +++ |
| 10 | Native Tragacanth | 160 | $<0 \cdot 01$ | +++ |
| 15 | Trag. heated for 30 min., 100° C | 80 | 0·53 | — |
| 15 | Trag., deionised | 120 | 0·06 | ++ |
| 10 | Ethylene glycol ester of Trag. | 200 | 1·00 | — |
| 10 | Reduced ethylene glycol ester of Trag. | 200 | 1·00 | — |
| 10 | Trag. ppted. from water by acetone | 200 | 0·84 | — |
| 10 | Gum Karaya (kutira) grade) | 400 | 0·94 | — |
| 5 | Gum Karaya (kutira) | 800 | 0·86 | — |

+++, strongly tumour-inhibitory (T/C, $<0 \cdot 05$);
++, inhibitory (T/C, 0·05 to 0·10);
—, non-tumour-inhibitory (T/C, $>0 \cdot 50$).

*Results*

From Fig. 1 and Table I it is seen that 80 or 160 mg./kg. doses of native Trag. strongly inhibit tumour growth, while inhibition by deionised Trag. is somewhat lower. Heated Trag. has an extremely low tumour-inhibitory activity; and other samples used in this study show no significant tumour-inhibition. Most samples had no adverse effects on the body weight of the mice. The exceptions were the heated Trag., deionised Trag. and the high dose of gum Karaya, all of which were slightly toxic, producing 1 g. weight loss per mouse in one week. The high (160 mg./kg.) dose of native Trag. produced a larger weight loss (2 g.). Under otherwise similar conditions, gum Karaya had no effect on the growth of the tumour, even at a dose ten times the optimum inhibitory dose of native Trag.

II. *Measurements of Mitotic Index*

Groups of B alb. C + ♂ mice bearing 7-days-old Landschütz ascites tumour were injected intraperitoneally with various Trag. samples, each suspended in 0·5 ml. isotonic saline. The doses used are shown in Table II. Control animals received saline only. At selected intervals after injection of the agent tumour cell samples were extracted from the mice by means of sterile syringes. Using aceto-orcein staining, the number of ascites tumour cells in mitosis was determined

TABLE II.

| Hours after injection | Control (saline) | Trag. (native) 80 mg./kg. | Trag. (native) 160 mg./kg. | Trag. (heated) 80 mg./kg. | Trag. (deionised) 80 mg./kg. | Trag. (ester) 80 mg./kg. | Trag. (reduced ester) (80 mg./kg. | Trag. (acetone pptd.) 80 mg./kg. | Gum karaya 80 mg./kg. |
|---|---|---|---|---|---|---|---|---|---|
| | Mitotic indices | | | | | | | | |
| 0 | 1·75 | 1·70 | 1·70 | 1·70 | 1·60 | 1·65 | 1·55 | 1·85 | 1·75 |
| 1 | 1·80 | 1·80 | 1·85 | — | — | — | — | — | — |
| 2 | 1·75 | 1·90 | 1·95 | 1·60 | 1·75 | 1·55 | 1·60 | 1·60 | 1·70 |
| 3 | 1·65 | 0·55 | 0·50 | — | — | — | — | — | — |
| 4 | 1·50 | 0·40 | 0·30 | 0·80 | 0·50 | 1·20 | 1·40 | 1·85 | 1·40 |
| 5 | 1·45 | 0·25 | 0·20 | — | — | — | — | — | — |
| 6 | 1·35 | 0·10 | 0·10 | — | — | — | — | — | — |
| 7 | 1·55 | 0·25 | 0·10 | — | — | — | — | — | — |
| 8 | 1·75 | 0·20 | 0·05 | 0·80 | 0·75 | 1·15 | 1·50 | 1·00 | 1·55 |
| 24 | 1·70 | 0·15 | 0·00 | 1·05 | 0·20 | 1·70 | 1·90 | 1·10 | 1·75 |
| 48 | 1·65 | 0·25 | — | 1·25 | 0·40 | 1·65 | 1·70 | 1·60 | 1·70 |
| 72 | 1·65 | 0·30 | — | 1·60 | 1·05 | 1·70 | 1·55 | 1·65 | 1·65 |
| 96 | 1·55 | 0·40 | — | 1·60 | 1·55 | 1·70 | 1·70 | 1·65 | 1·50 |
| 120 | 1·55 | 0·55 | — | 1·55 | 1·65 | 1·70 | 1·75 | 1·55 | 1·60 |

from a sample of 4000 tumour cells on each slide. The percentage of these in mitosis (i.e. the total mitotic index) was calculated from the pooled results from two animals used at each dose and time after treatment. For tumours treated with 80 mg./kg. Trag., an extra count was made, i.e. in 100 dividing cells on each slide the stage of mitosis was noted so that a calculation of the percentage of ascites tumour cells in each stage of mitosis was possible. The anaphase and telophase totals were individually too small to enable significant comparisons to be made with the other mitotic stages. So these totals were added together. In cases where the mitotic index was very greatly reduced it was impossible

FIG. 1.—Growth inhibition of Landschütz ascites tumour after intraperitoneal treatment with a single dose of native gum tragacanth in saline. (Each point on graph is a mean result from five mice.)

to find 200 dividing cells on the two slides. These cases are noted in Table III. No result was recorded if fewer than 50 dividing cells were found.

Preliminary determinations of the statistical significance of the results were made. These indicated that a difference of more than $\pm$ 5 per cent (i.e. more than $\pm$ 0·05 for a mitotic index of 1·0) could be considered significant ($P < 0{\cdot}05$). The significance levels adopted in this work for the differences at various stages of mitotis were : prophase, more than $\pm$ 15 per cent ; metaphase, more than $\pm$ 10 per cent ; and anaphase plus telophase, more than $\pm$ 15 per cent.

*Results*

The results of these mitotic index measurements are shown in Table II. It will be noticed that there are small but significant changes in the mitotic indices of the control tumours. There appeared to be both a daily mitotic cycle and also a gradual decrease in mitotic index as the tumour aged. These phenomena were

TABLE III.

| Hours after injection | Mitotic Indices: Prophase Control | Prophase Trag., 80 mg./kg. | Metaphase Control | Metaphase Trag., 80 mg./kg. | Anaphase plus telophase Control | Anaphase plus telophase Trag., 80 mg./kg. |
|---|---|---|---|---|---|---|
| 0 | 0·49 | 0·47 | 0·88 | 0·83 | 0·38 | 0·39 |
| 1 | 0·38 | 0·39 | 0·79 | 0·88 | 0·63 | 0·53 |
| 2 | 0·46 | 0·51 | 0·91 | 1·12 | 0·38 | 0·27 |
| 3 | 0·52 | 0·19 | 0·86 | 0·25 | 0·30 | 0·10 |
| 4 | 0·38 | 0·14 | 0·78 | 0·20 | 0·34 | 0·06 |
| 5 | 0·48 | 0·07* | 0·70 | 0·13* | 0·28 | 0·03* |
| 6 | 0·47 | 0·03* | 0·68 | 0·05* | 0·21 | 0·02* |
| 7 | 0·46 | 0·09* | 0·78 | 0·12* | 0·28 | 0·03* |
| 8 | 0·61 | 0·07* | 0·82 | 0·10* | 0·31 | 0·03* |
| 24 | 0·51 | 0·05* | 0·87 | 0·08* | 0·32 | 0·02* |
| 48 | 0·50 | 0·08 | 0·79 | 0·15 | 0·36 | 0·04 |
| 72 | 0·54 | 0·09* | 0·79 | 0·16* | 0·31 | 0·05* |
| 96 | 0·50 | 0·10 | 0·81 | 0·21 | 0·25 | 0·09 |
| 120 | 0·42 | 0·14 | 0·73 | 0·27 | 0·40 | 0·14 |

* Less than 200 dividing cells observed.

eliminated in the assessment of the effects of gum treatment, by expressing the mitotic index of the treated tumour cells as a percentage of that of the controls and plotting this figure against the time after treatment (Fig. 2A, 2B and 3). If treatment had no effect on the mitotic index then the plot would lie parallel to the abscissa at the 100 per cent mark. It can be seen from the figures that initially most gum samples have some effect on the mitotic index, but that divergences soon appear. The effects of the various agents will be discussed separately.

(a) Native Trag., 160 mg./kg. dose (Fig. 2A) : This dose caused a small but significant rise in the total mitotic index during the first two hours after treatment, but within the next hour the index dropped sharply and this reduction continued until 24 hours after treatment when 100 per cent inhibition was achieved. No tumour could be extracted at later times.

(b) Native Trag., 80 mg./kg. dose (Fig. 2A) : After this dose a small early rise in the mitotic index occurred as in (a) above, and again a sharp drop followed, to give a mitotic index of 30 per cent of the control value after 3 hours' treatment and 7 per cent after six hours. No further change occurred up to 24 hours after treatment, when a slow rise in the mitotic index commenced reaching 35 per cent of the control value after five days.

Table III and Fig. 3 show the results of mitotic index measurements at different stages of mitosis after the 80 mg./kg. dose of native Trag. It can be seen that changes in the prophase index followed the total mitotic index changes throughout the experiment. However, at one and two hours after treatment there was a rise in the index of cells at metaphase and a correspondingly reduced index for those at anaphase. These results could arise if, during this period, the number of cells reaching prophase was not significantly altered, but cells reaching metaphase were unable to pass to anaphase. It also follows, from this decrease in the anaphase index, that cells reaching anaphase and telophase were able to complete division.

FIG. 2A.—Effect on mitotic index (expressed as percentage of controls) of Landschütz ascites tumour of i.p. treatment: native tragacanth (dose/mouse, 80 mg./kg.) –.–.–. ; native tragacanth (160 mg./kg.) ...... ; heat deactivated tragacanth (80 mg./kg.) ——— ; gum karaya (80 mg./kg.) – – – – –.

FIG. 2B.—As 2A :—deionised tragacanth (80 mg./kg.) ——— ; ethylene glycol ester of deionised tragacanth (80 mg./kg.) ...... ; reduced ethylene glycol ester of deionised tragacanth (80 mg./kg.) – – – – – – ; tragacanth ppted. from aqueous suspension with acetone (80 mg./kg.) — — —.

(c) Trag. heated at 100° C. for 30 minutes, 80 mg./kg. dose (Fig. 2A) : After this treatment the total mitotic index dropped gradually, reaching a minimum value (45 per cent) after 8 hours. Tumour recovery then commenced so that, 72 hours after treatment, the total index had reached the control value.

(d) Gum karaya, 80 mg./kg. dose (Fig. 2A) : This gum, sometimes used as a substitute for Trag. in pharmacy, has been shown to be non-inhibitory. It produced a small decrease in the mitotic index (to 90 per cent of the control value) eight hours after treatment but at later times had no significant mitotic effect.

(e) Deionised Trag., 80 mg./kg. dose (Fig. 2B) : A small initial rise in the mitotic index occurred, as with native Trag., followed by a steady fall to 33 per cent

FIG. 3.—Effect on different stages of mitosis of Landschütz ascites tumour cells of i.p. treatment with native tragacanth (dose/mouse, 80 mg./kg. ; number of cells at each stage expressed as percentage of corresponding control) : total mitotic index ——— ; prophase index — — — ; metaphase index - - - - - - ; anaphase plus telophase indices . . . . . .

of the control value after 4 hours and to 10 per cent after 24 hours. Slow recovery then occurred, reaching approximately the control value 96 hours after treatment.

(f) Ethylene glycol ester of deionised Trag., 80 mg./kg. dose (Fig. 2B) : Using this sample, a fall in the mitotic index (to a value of 65 per cent of controls) was noted during the first 8 hours after treatment, followed by recovery to the control value 24 hours after treatment.

---

EXPLANATION OF PLATE

Fig. 4. —A. Landschütz ascites tumour cell controls stained by PAS after treatment *in vivo* with isotonic saline. B. Similar PAS-stained cells, 10 minutes after *in vivo* treatment with native tragacanth (80 mg./kg.).

4

Mayhew and Roe.

(g) Reduced ethylene glycol ester of deionised Trag., 80 mg./kg. dose (Fig. 2B): This treatment produced no significant difference between the mitotic index values for control and treated animals at any time.

(h) Trag. precipitated with acetone, 80 mg./kg. dose (Fig. 2B): This sample produced changes somewhat similar to those resulting from treatment with the ester (f) above, but with a sharper fall in mitotic index (to 60 per cent in 4 hours) and a more gradual recovery, after 8 hours, to the control value.

### DISCUSSION

It will be noted, from the results described, that gum samples which significantly inhibit ascites tumour growth cause an increase in the total mitotic index of the tumour 1 to 2 hours after treatment, followed later by a fall in the mitotic index to 10 per cent or less of the control value. Recovery in the mitotic index, if it occurs, is slow. There is no initial rise in the mitotic index of the tumour after treatment with non-inhibitory gum samples. Further, any fall in mitotic index which occurs in these cases is much less and recovery to the control level is more rapid than after treatment with the inhibitory gums.

The initial increase in total mitotic index of tumours treated with tumour-inhibitory gum samples is due to an increase in the number of cells at metaphase. Further, the reduction in anaphase mitotic index reveals the inability of many cells to proceed to anaphase. Thus, Trag. inhibits mitosis during the first two hours after treatment; three hours and later after treatment there is no observable mitotic inhibition but the number of tumour cells at each stage of division is considerably reduced. This reduction in total mitotic index could indicate (a) that fewer cells reach division; or (b) that all cells reach division but pass through it more quickly Since it has been shown that the number of ascites tumour cells present per mouse decreases after Trag. treatment the second possibility (b) can be eliminated, demonstrating that possibility (a) above, i.e. cytostasis, is also a result of the treatment with native Trag.

There is no evidence that the two consecutive effects of the native gum, mitotic inhibition and cytostasis, are distinct modes of action. They may be effects on cells which are at different stages in the cell cycle at the time of injection of the agent, i.e. in interphase or in division. Since measurements of mitotic index record only the cells which are in division, a mitotic block would be observed earlier than the effects of treatment on the interphase cells initially present. The latter will be recorded in division some hours after treatment begins and the cytostasis observed may be an effect on these resting cells.

The results presented in this paper show that native Trag. affects the mitosis of Landschütz ascites tumour cells within one hour of its injection into the peritoneal cavity. In earlier *in vitro* studies (Galbraith, Mayhew and Roe, 1962) it was observed that the Trag. became attached to the surfaces of the tumour cells during this time and more recently (Mayhew and Roe, unpublished) this observation has been confirmed *in vivo*. The latter experiments showed that the native Trag. or a complex of Trag. with components of the ascitic fluid becomes attached to the tumour cell surface during the first ten minutes after injection (Fig. 4B) and remains there for at least 5 to 6 hours. No observable penetration of Trag. into the cells occurs until later times. It is concluded, therefore, that the growth-inhibitory action of native Trag. against these ascites tumour cells is due to metabolic damage, revealed by mitotic inhibition and cytostasis, and that this damage is initiated by the attachment of the active component of the gum to the cell surface during the first hour of treatment.

### SUMMARY

The tumour-inhibitory and mitotic effects, on Landschütz ascites tumour cells, of native gum tragacanth (high grade Persion ribbon) of various modified samples of this gum, and of gum karaya have been compared. Tumour-inhibitory gum samples affect mitosis within one hour after their injection intraperitoneally, producing a mitotic block at metaphase, and, later, cytostasis.

The authors are grateful to Professor A. Haddow, F.R.S., for his encouragement in this work, and to Dr. R. Ferrier, of Birbeck College, London, for his collaboration in the chemical treatment of the gum tragacanth.

This investigation has been supported by grants to the Chester Beatty Research Institute (Institute of Cancer Research : Royal Cancer Hospital) from the Medical Research Council, the British Empire Cancer Campaign for Research, and the National Cancer Institute of the National Institutes of Health, U.S. Public Health Service.

### REFERENCES

Galbraith, W., Mayhew, E. and Roe, E. M. F.—(1962) *Brit. J. Cancer*, **16**, 163.
Roe, E. M. F.—(1959) *Nature, Lond.*, **184**, 1891.
Tjio, J. H. and Levan, A.—(1954) *Acta Univ. lund.*, Avd. 2, 50, No. 15.